# Extending the Lower Limits of Body Weight for Bi-directional Glenn Shunt in Developing Countries: Is it Feasible?

**DOI:** 10.7759/cureus.118383

**Published:** 2026-10-22

**Authors:** Sachin Talwar, Debasis Das, Vishal V Bhende, Anjani A Gohil, Mathangi Krishnakumar, Ashwin S Sharma

**Affiliations:** 1 Cardiothoracic and Vascular Surgery, All India Institute of Medical Sciences, New Delhi, New Delhi, IND; 2 Cardiothoracic Surgery, Narayana Health - Narayana Superspeciality Hospital, Kolkata, IND; 3 Pediatric Cardiac Surgery, Bhanubhai and Madhuben Patel Cardiac Centre, Bhaikaka University, Karamsad, IND; 4 Pediatric Cardiac Surgery, Sri Sathya Sai Sanjeevani Centre for Child Heart Care and Training in Pediatric Cardiac Skills, Navi Mumbai, IND; 5 Surgery, Pramukhswami Medical College, Shree Krishna Hospital, Bhaikaka University, Karamsad, IND; 6 Surgery, New Civil Hospital, Surat, IND; 7 Anaesthesiology, St.John's Medical College Hospital, Bengaluru, IND; 8 Internal Medicine, Gujarat Cancer Society Medical College, Hospital and Research Centre, Ahmedabad, IND

**Keywords:** bidirectional glenn shunt, cardiopulmonary bypass, complex congenital heart disease, low weight-for-age, single-ventricle physiology

## Abstract

The bi-directional Glenn (BDG) shunt is an established stage of palliation for functionally single-ventricle congenital heart disease, but optimal timing and the influence of severe underweight remain uncertain. We report a 102-day-old male infant, born at 38 weeks' gestation and weighing 2.7 kg at surgery, with double-outlet right ventricle, difficult/non-routable intracardiac anatomy, D-malposed great arteries, and severe pulmonary stenosis who underwent a right-sided BDG with atrial septectomy and partial main pulmonary artery (PA) ligation. The infant was discharged on postoperative day 11 with an oxygen saturation of 92%. Available follow-up documentation over 18 months described a patent BDG on two-dimensional echocardiography. This single case demonstrates technical feasibility in a selected severely underweight infant; it does not establish a safe lower age or weight threshold, comparative mortality, or long-term Fontan outcome. Larger studies with complete nutritional, hemodynamic, and longitudinal follow-up data are required.

## Introduction

The bi-directional Glenn (BDG) shunt is an established intermediate palliative procedure for patients with functionally single-ventricle physiology before Fontan completion [1]. Earlier cavopulmonary connection may reduce ventricular volume loading and avoid some consequences of prolonged systemic-to-pulmonary shunt physiology, but patient selection remains dependent on anatomy, pulmonary vascular physiology, ventricular and atrioventricular valve function, and the adequacy of the pulmonary arteries [2-4]. Primary BDG shunt in selected infants as young as one to four months has been reported previously, including patients with double-outlet right ventricle; therefore, young age alone is not a novel indication or an established contraindication [2]. Earlier reports described the role and longer-term experience of cavopulmonary connections [5,6], while subsequent work addressed mechanical efficiency and timing within staged single-ventricle palliation [7,8].

Growth failure is common in infants with single-ventricle physiology and may influence perioperative recovery. Anderson et al. reported that lower weight-for-age z-score (WAZ) was associated with longer postoperative hospitalization after BDG, and a developing-country cohort likewise identified low WAZ and younger age as risk factors for prolonged postoperative mechanical ventilation [9,10]. Recent Indian observational data also emphasize that age and operative context should be interpreted together rather than as isolated thresholds [11]. The specific contribution of the present report is the successful performance of BDG in one markedly underweight infant weighing 2.7 kg at 102 days of age. We therefore describe this case as an observation of individual technical feasibility rather than evidence for a new lower weight or age limit.

## Case presentation

A 102-day-old male infant weighing 2.7 kg underwent a BDG procedure at Bhanubhai and Madhuben Patel Cardiac Centre, Karamsad, on May 22, 2020. He was born at 38 weeks' gestation with a birth weight of 2.25 kg and weighed 2.38 kg at neonatal discharge on day six of life. Archived preoperative records document poor weight gain, severe cyanosis with a history suggestive of cyanotic spells, breastfeeding with supplemental 'top' feeds, and dyspnea for 15-20 days before the cardiac computed tomography(CT). On May 16, 2020, preoperative echocardiography recorded a weight of 2.5 kg, recumbent length of 49 cm, and oxygen saturation of 56%.

Echocardiography and cardiac CT demonstrated double-outlet right ventricle (DORV) with >50% aortic override, a large/subaortic ventricular septal defect, D-malposed great arteries, and severe valvar/subvalvar pulmonary stenosis (reported peak right ventricular outflow gradient 68 mmHg). The right atrium and right ventricle were dilated; ventricular systolic function was described qualitatively as good; there was mild tricuspid regurgitation and no mitral regurgitation. The superior and inferior venae cavae drained to the right atrium, and all four pulmonary veins drained normally to the left atrium. Cardiac CT showed mildly hypoplastic pulmonary arteries: proximal/distal right pulmonary artery (RPA) 4.4/4.5 mm and proximal/distal left pulmonary artery (LPA) 4.7/6.0 mm, with a McGoon ratio of 1.40 and Nakata index of 377.37 mm^2^/m^2^. No patent ductus arteriosus or major aortopulmonary collateral was identified. No previous systemic-to-pulmonary shunt was documented. Intraoperative assessment during atrial septectomy confirmed difficult intracardiac anatomy considered unsuitable for biventricular routing, and the patient was therefore managed along a functionally single-ventricle pathway with primary BDG palliation. The records do not document respiratory support before surgery, the formal urgency category, or a separate rationale for proceeding to surgery rather than further nutritional optimization. The retrospective case report received ethical approval from the Institutional Ethics Committee (IEC-2) of the HM Patel Centre for Medical Care and Education, Anand, Gujarat (Approval No. IEC/HMPCMCE/2020/Cr. 12/177/20; July 27, 2020).

Written informed consent for publication of the infant's clinical information and images was obtained from a parent/legal guardian. The July 27, 2020 institutional approval pertained to retrospective case reporting and was distinct from consent for the clinical treatment performed in May 2020.

Preoperative evaluation included biochemical investigations, echocardiography, cardiac CT, and a preoperative chest radiograph (Figure 1). The postoperative chest radiograph is shown separately in Figure 2.

**Figure 1:**
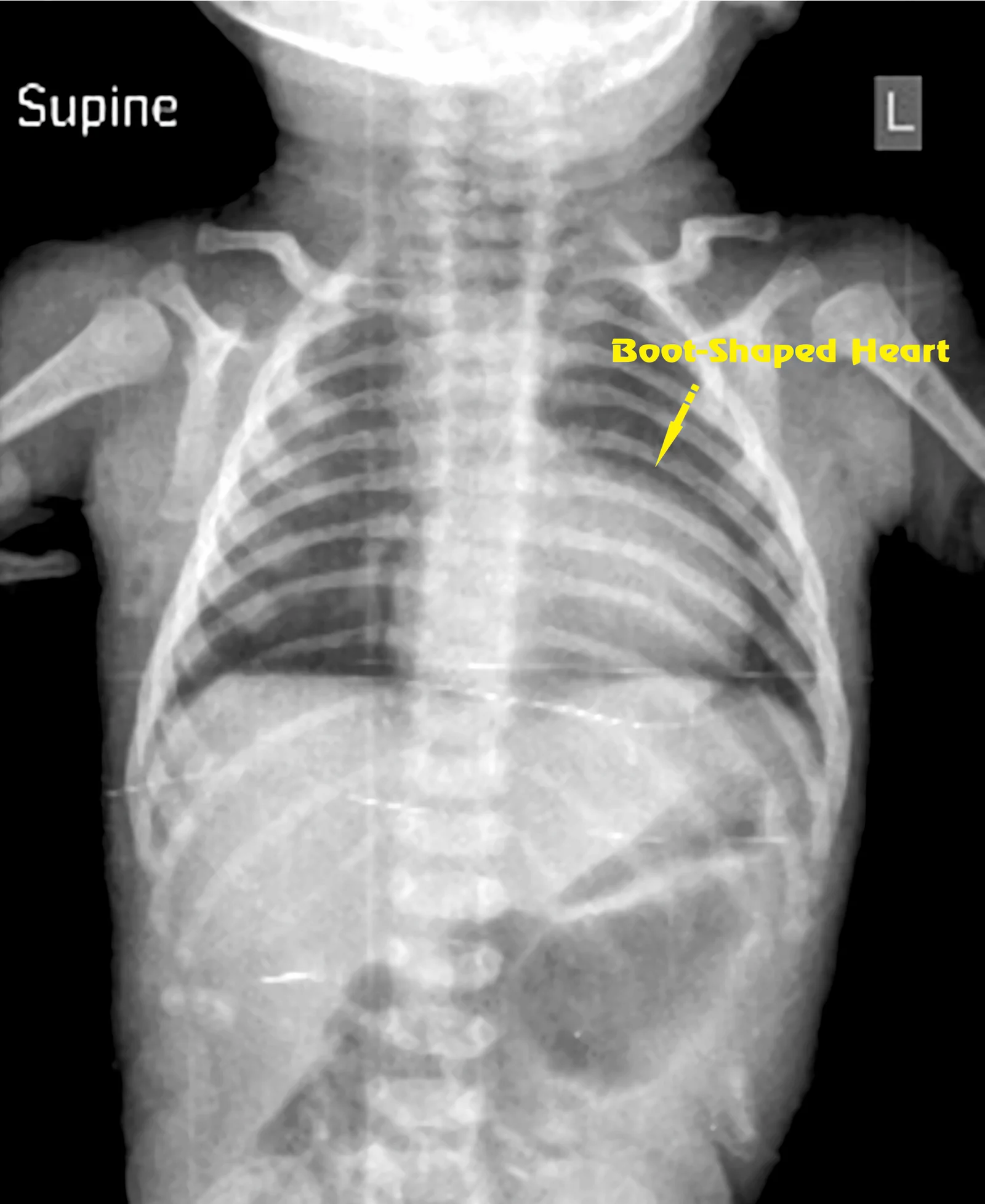
Pre-operative chest radiograph obtained during assessment before the BDG procedure. The precise interval between radiography and surgery was not available in the source documentation. BDG: bi-directional Glenn.

**Figure 2:**
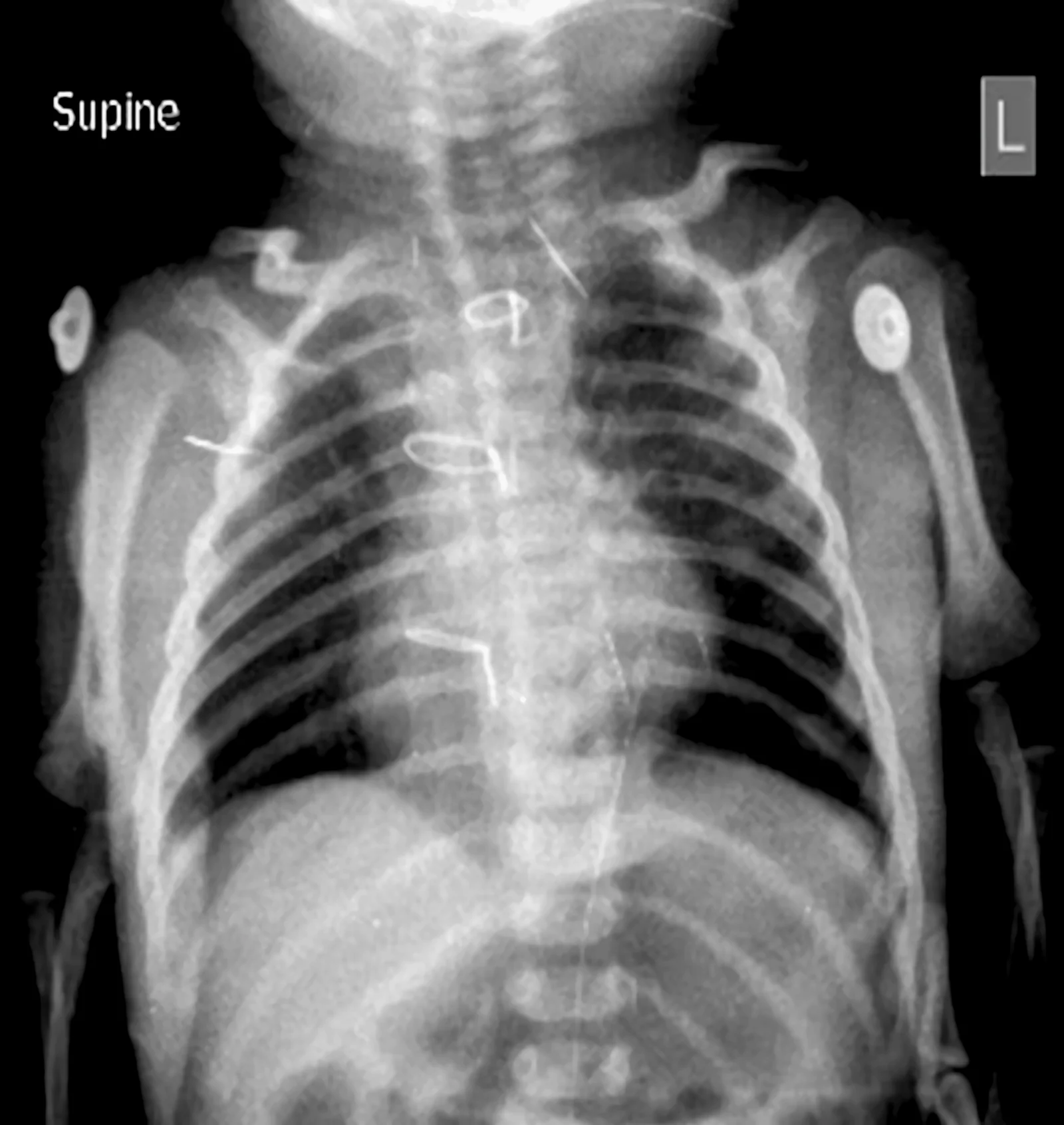
Post-operative chest radiograph after the BDG procedure. The precise post-operative day was not available in the source documentation. BDG: bi-directional Glenn.

Before cardiopulmonary bypass, pulmonary artery (PA) pressure was measured directly by inserting a 26-G needle into the RPA and was 16/8 mmHg (mean 11 mmHg). No preoperative cardiac catheterization was performed, and pulmonary vascular resistance was not formally measured; the ventilatory conditions and atrial/filling pressures at the time of the direct PA pressure measurement were not available. Suitability for BDG was therefore assessed from the clinical, echocardiographic, CT, and intraoperative findings rather than from catheter-derived pulmonary vascular resistance (PVR). A right-sided BDG was completed with atrial septectomy and partial main pulmonary artery (MPA) ligation. The specific rationale for the atrial septectomy and degree of MPA ligation was not separately documented in the archived operative material. However, the immediate postoperative echocardiogram documented no forward flow across the pulmonary valve and described the BDG as non-pulsatile, indicating no appreciable residual antegrade pulmonary flow at that assessment. Post-bypass SVC/PA pressures were not available (Figures 3, 4).

**Figure 3:**
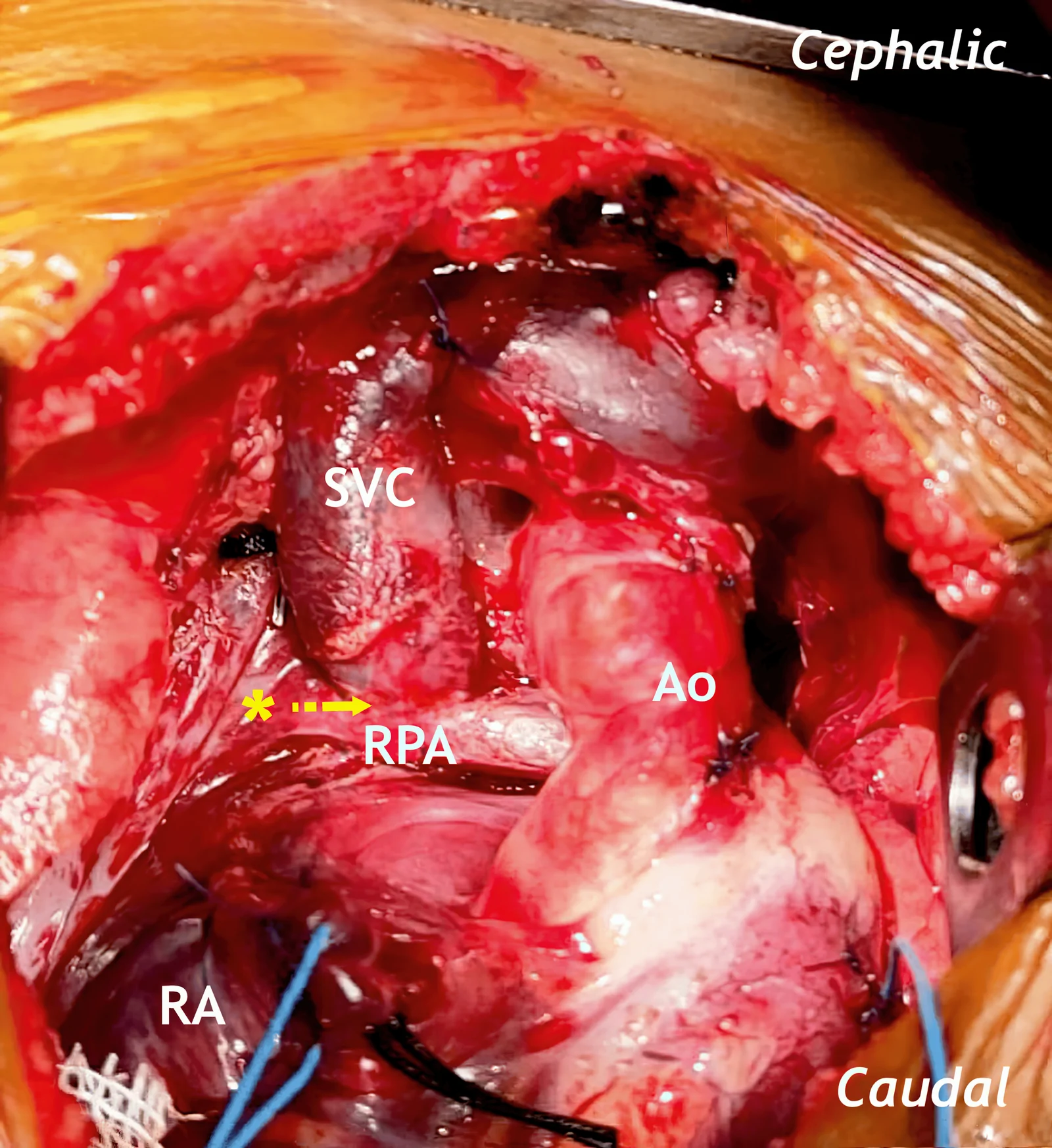
Completed BDG shunt procedure. RPA: right pulmonary artery; RA: right atrium; SVC: superior vena cava; AO: aorta; * (Asterisk) facing the arrow denotes Glenn anastomosis; BDG: bi-directional Glenn.

**Figure 4:**
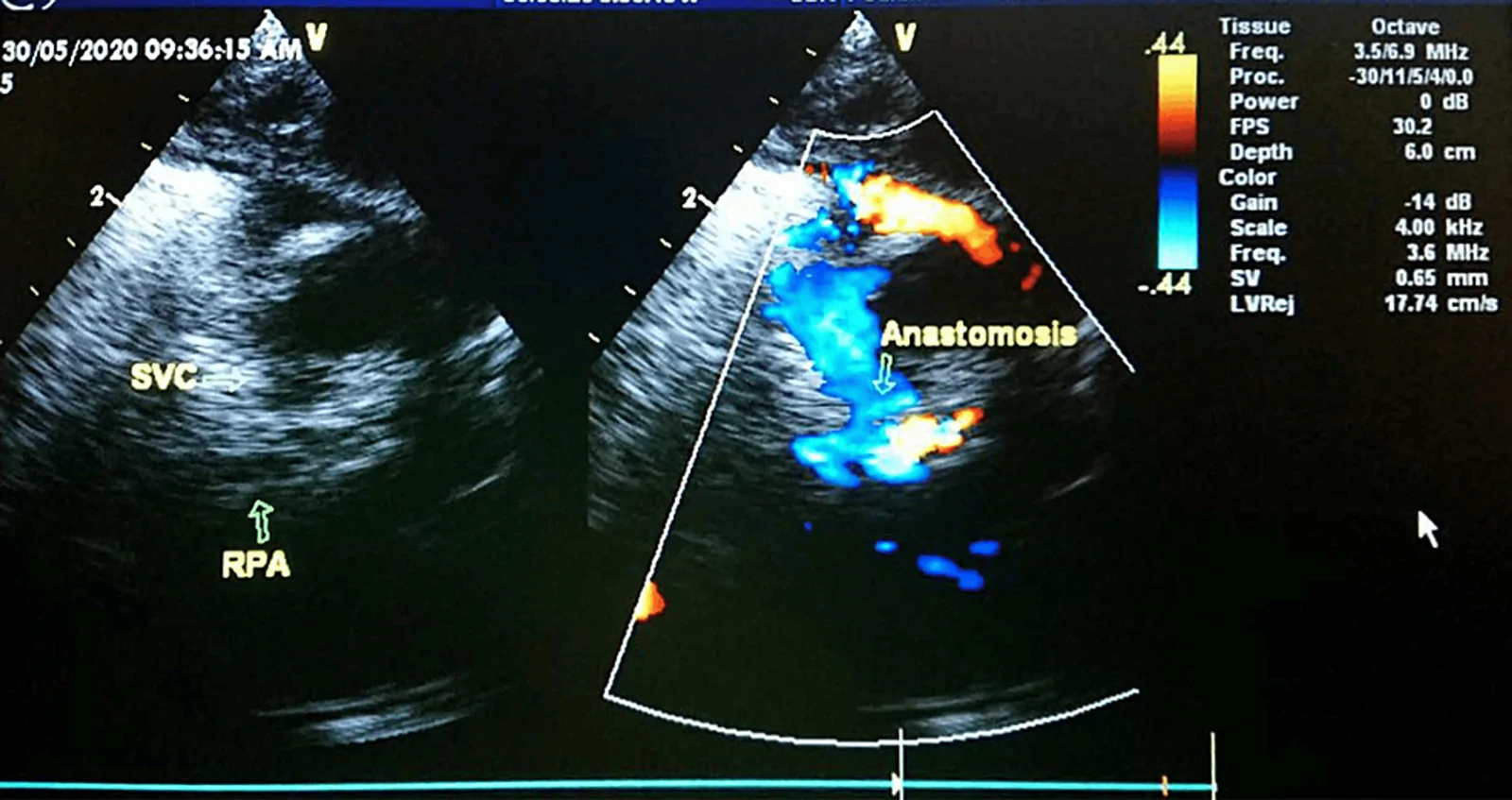
Two-dimensional/color-flow echocardiographic image of the BDG anastomosis between the SVC and right RPA. This still image documents anatomy and color flow but does not display the spectral Doppler trace used for gradient measurement. BDG: bi-directional Glenn; SVC: superior vena cava; RPA: right pulmonary artery.

Direct laryngoscopy was documented as Modified Cormack-Lehane grade 4 [12]. The archived records do not document the rescue airway device, number of attempts, or adjuncts used, and these details are therefore not inferred. A syndromic phenotype raised clinical suspicion of 22q11.2 deletion syndrome; genetic testing was not performed, so this remained a clinical suspicion rather than a confirmed diagnosis. Anthropometric assessment was undertaken during the neonatal admission and at the time of BDG (Figures 5, 6).

**Figure 5:**
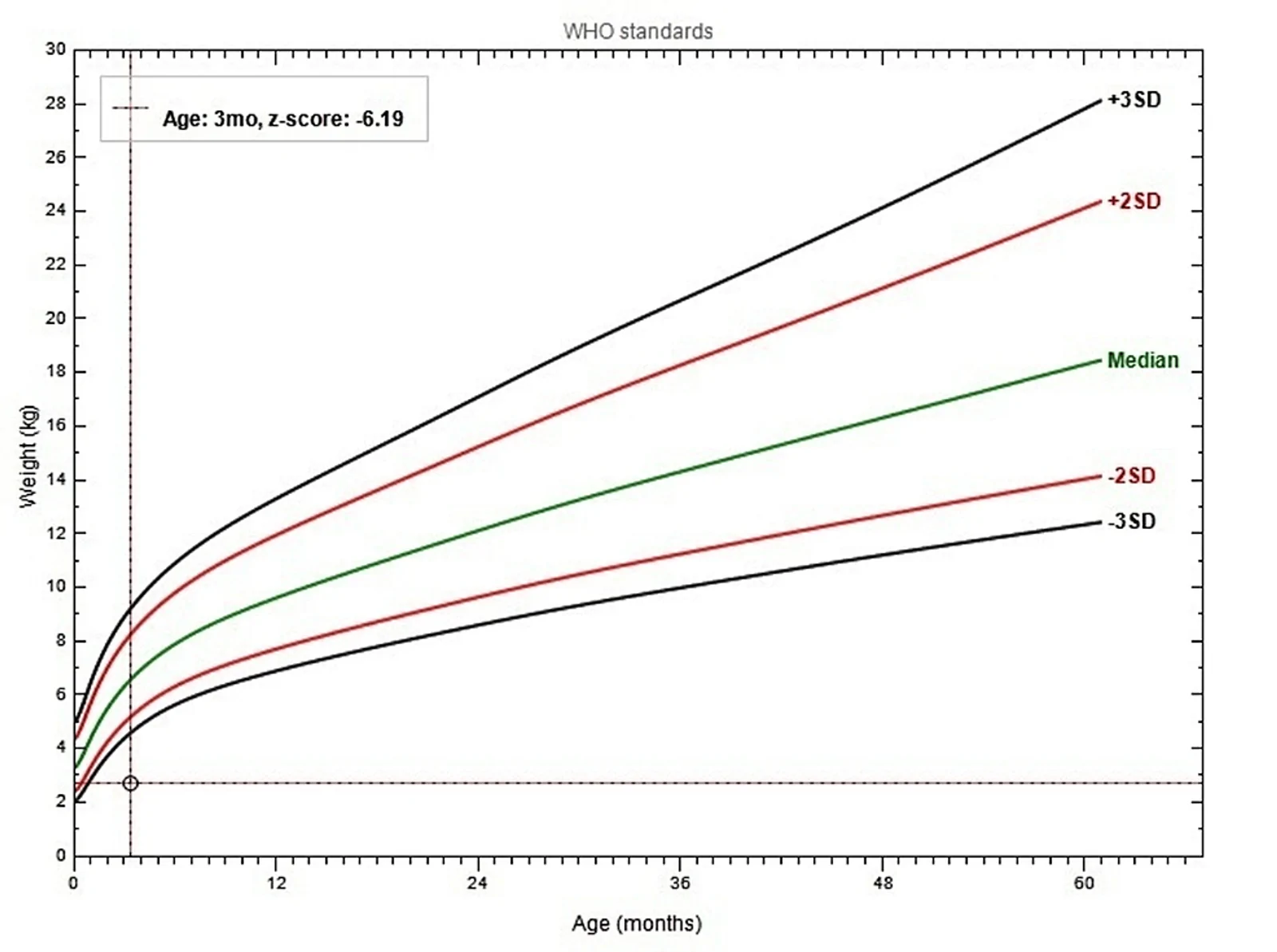
Reported WHO Anthro weight-for-age assessment at the BDG procedure in a male infant aged 102 days and weighing 2.7 kg. The plotted 3-month label is a display label; the manuscript reports the exact chronological age. BDG: bi-directional Glenn.

**Figure 6:**
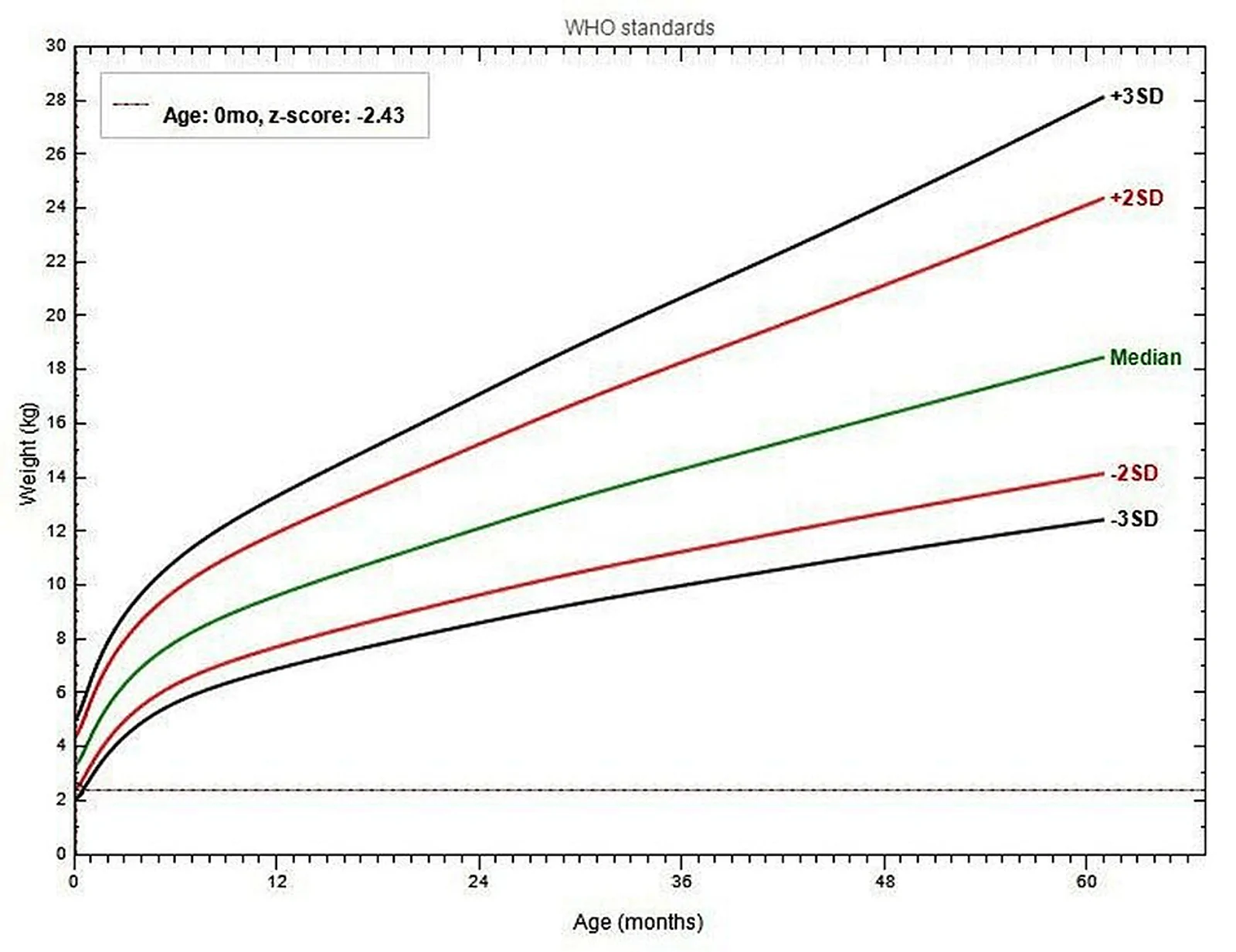
Reported WAZ in this male infant: -2.43 at 6 days of life (weight 2.38 kg) and -6.19 at the BDG procedure at 102 days (operative weight 2.7 kg). The infant was born at 38 weeks' gestation, so no prematurity correction was applied. WAZ: weight-for-age z score; BDG: bi-directional Glenn.

WAZs were reported using WHO Anthro [13]. The infant was male, born at 38 weeks' gestation with a birth weight of 2.25 kg; no prematurity correction was therefore applied. He weighed 2.38 kg at six days of life and 2.7 kg at the 102-day BDG procedure. A preoperative recumbent length of 49 cm was documented on May 16, 2020. The reported WAZ values were -2.43 on day six and -6.19 at BDG. Comparison with the sex-specific WHO weight-for-age standards confirms that the day-six weight was below -2 standard deviations (SD) and the operative weight was far below -3 SD; the exact two-decimal values are retained from the original WHO Anthro output because the software version and original electronic calculation file were not preserved. The ages shown as 0 and three months in the figures are display labels; the chronological age at surgery was 102 days. The infant had poor weight gain and was receiving breast feeds with supplemental feeds before surgery. WAZ is used here specifically to describe underweight-for-age; wasting and stunting are not inferred from WAZ alone.

The immediate postoperative echocardiogram described a well-flowing right-sided non-pulsatile BDG, a peak/mean Doppler gradient of 4/2 mmHg across the superior vena cava-right pulmonary artery (SVC-RPA) anastomosis, RPA and LPA diameters of 4.2 and 4.0 mm, respectively, no forward flow across the pulmonary valve, good biventricular systolic function, mild tricuspid regurgitation, and no mitral regurgitation; the recorded oxygen saturation at that study was 72%. The infant was discharged on postoperative day 11 with stable hemodynamics and an oxygen saturation of 92%; oxygen-support status at discharge was not specified in the archived record. The 4/2 mmHg gradient is interpreted only as an anastomotic Doppler flow measurement and not as a measure of ventricular function; the available Figure 4 is a two-dimensional/color-flow still image and does not display the spectral Doppler trace. Follow-up documentation over 18 months described continued patency of the right-sided BDG on two-dimensional echocardiography. Detailed serial oxygen saturation, weight/WAZ, feeding status, ventricular/valve measurements, branch PA growth, thrombosis, venous hypertension, chylothorax, readmissions, reinterventions, discharge thromboprophylaxis, developmental assessment, and later Fontan evaluation/plan were not available in the archived material. The 18-month observation is therefore not presented as evidence of completed Fontan palliation or durable long-term outcome (Tables 1, 2, 3).

**Table 1:** Peri-operative data. BDG: bi-directional Glenn; PA: pulmonary artery; RPA: right pulmonary artery; MPA: main pulmonary artery; CPB: cardiopulmonary bypass; ACC: aortic cross-clamp; CT: computed tomography; LPA: left pulmonary artery; PVR: pulmonary vascular resistance; AV: atrioventricular.

Sr. No.	Parameters	Patient Values
01	Age during the BDG procedure	102 days
02	Weight at the time of the BDG procedure	2.7 kg
03	Intraoperative pre-bypass PA pressure	16/8 mmHg (mean 11), measured directly pre-bypass with a 26-G needle in the RPA; ventilatory conditions not available
04	Associated procedures performed	Atrial septectomy; MPA partial ligation
05	CPB time	93 min.
06	ACC time	53 min.
07	Branch pulmonary-artery dimensions/indexed values	CT: RPA 4.4/4.5 mm (proximal/distal), LPA 4.7/6.0 mm; McGoon ratio 1.40; Nakata index 377.37 mm^2^/m^2^
08	Preoperative catheterization/PVR/filling pressures	No cardiac catheterization; PVR not formally measured; filling pressures not available
09	Preoperative ventricular/AV-valve assessment	Good biventricular systolic function; mild tricuspid regurgitation; no mitral regurgitation
10	Preoperative oxygen saturation	56% on echocardiography dated May 16, 2020

**Table 2:** Post-operative data. SICU: surgical intensive care unit; BDG: bi-directional Glenn; RPA: right pulmonary artery; LPA: left pulmonary artery; TR: tricuspid regurgitation; MR: mitral regurgitation.

Sr. No.	Parameters	Patient Values
01	Mechanical ventilatory support time	48 hours
02	Chest tube duration	68 hours (3 days)
03	Duration of SICU stay	5 days
04	Duration of hospital stay	11 days
05	Discharge oxygen saturation	92% on postoperative day 11; oxygen-support status not specified
06	Documented follow-up duration	18 months
07	BDG anastomotic Doppler gradient	4/2 mmHg (peak/mean) on postoperative echocardiography dated May 30, 2020; spectral trace not available in Figure 4
08	Immediate postoperative echocardiographic findings	Well-flowing non-pulsatile right-sided BDG; RPA 4.2 mm, LPA 4.0 mm; no forward pulmonary-valve flow; good biventricular function; mild TR; no MR; recorded saturation 72%
09	Detailed serial 18-month outcome/Fontan data	Not available beyond documentation of BDG patency

**Table 3:** Clinical timeline from neonatal presentation through the available follow-up. NICU: neonatal intensive care unit; WAZ: weight-for-age z-score; DORV: double-outlet right ventricle; TR: tricuspid regurgitation; CT: computed tomography; PA: pulmonary artery; BDG: bi-directional glenn shunt; MPA: main pulmonary artery.

Date/age	Weight	Documented clinical/imaging information	Outcome
10 Feb 2020 (birth)	2.25 kg	Male infant, 38 weeks' gestation; neonatal record documents cyanotic congenital heart disease	NICU admission
16 Feb 2020 (day 6)	2.38 kg	Neonatal discharge; reported WAZ -2.43	Discharged from neonatal admission
24 Feb 2020 (14 days)	2.36 kg	Outpatient record; head circumference 31.2 cm	Follow-up
16 Mar 2020 (1 month 5 days)	2.52 kg	Outpatient review	Persistent poor weight gain
16 May 2020	2.5 kg	Length 49 cm; saturation 56%; DORV, severe pulmonary stenosis, good biventricular function, mild TR	Preoperative echocardiography
18 May 2020	2.5 kg	Dyspnea for 15-20 days; CT defined DORV anatomy, PA dimensions, McGoon 1.40, Nakata 377.37 mm^2^/m^2^	Preoperative cardiac CT
22 May 2020 (102 days)	2.7 kg	Primary right-sided BDG with atrial septectomy and partial MPA ligation	Surgery
30 May 2020	2.5 kg	Saturation 72%; well-flowing non-pulsatile BDG; gradient 4/2 mmHg; good biventricular function	Immediate postoperative echocardiography
Postoperative day 11	Not documented	Saturation 92%; stable hemodynamics	Hospital discharge
Available follow-up to 18 months	Serial weights not available	BDG described as patent on two-dimensional echocardiography; detailed serial measurements and Fontan plan unavailable	Follow-up limited to available documentation

## Discussion

This case documents the technical feasibility of a primary BDG in one selected 102-day-old infant weighing 2.7 kg. Primary cavopulmonary palliation in young infants has precedent: Reddy et al. described selected infants aged one to four months undergoing primary bi-directional superior cavopulmonary shunt [2], and subsequent series have examined early BDG in different anatomical and staged-palliation settings [3,4,14]. These reports do not establish a universal lower age or weight threshold; instead, they emphasize careful selection and the importance of pulmonary vascular and ventricular physiology. The present case adds an observation at an unusually low absolute weight, but it should not be interpreted as evidence that severe underweight is generally without consequence.

WHO growth standards use age- and sex-specific z-scores to describe weight-for-age [13]. In single-ventricle patients, nutritional status is clinically relevant: Anderson et al. found that lower WAZ was associated with longer postoperative hospitalization after BDG in 100 infants [9]. In a developing-country cohort, lower WAZ and younger age were also associated with adverse postoperative outcomes, including prolonged mechanical ventilation [10]. Accordingly, the favorable course in this individual patient is compatible with, rather than contradictory to, cohort-level associations between poor growth and morbidity. In this report, the documented male sex, exact ages and weights, and term gestation support classification as marked underweight-for-age. The original WHO Anthro software version and electronic calculation file were not preserved, so the exact two-decimal WAZ values are retained from the original output rather than independently recalculated. WAZ alone does not classify wasting or stunting.

The physiological rationale for BDG includes partial ventricular volume unloading by routing superior vena caval return directly to the pulmonary arteries. We removed the previous numerical comparison suggesting that a universal indexed cardiac-output requirement falls from 4 to 3 L/min because the cited studies examined different patient groups and physiological conditions and did not provide a paired threshold applicable to this patient. In this case, partial MPA ligation was performed, and the immediate postoperative echocardiogram documented no forward flow across the pulmonary valve, supporting the description of a non-pulsatile Glenn circulation at that assessment. The reported 4/2 mmHg anastomotic Doppler gradient is not used as evidence of ventricular function; ventricular systolic function was instead described separately as qualitatively good on echocardiography.

Interpretation is limited by the single-case design, selection and publication bias, incomplete longitudinal nutritional and hemodynamic follow-up, absence of catheter-derived PVR and post-bypass cavopulmonary pressure data, and unavailable details of the difficult-airway rescue strategy. Although the archived CT and echocardiographic records now provide objective branch pulmonary-artery dimensions, venous anatomy, baseline saturation, and qualitative ventricular/valve assessment, the report still lacks a comparator and comprehensive serial outcome measurements. It does not compare strategies, quantify healthcare utilization or cost, define a safe lower weight/age limit, establish mortality equivalence with older infants, or predict Fontan completion. Contemporary observational studies, including data from developing-country and Indian settings, show that age, nutritional status, anatomy, and perioperative physiology can influence outcomes [10,11]. The value of this case is therefore descriptive: it illustrates that BDG was technically achievable in one severely underweight infant under the reported clinical circumstances.

Published Indian experience includes BDG in infants. Talwar et al. described eight procedures within the first two months of life, with the youngest patient aged 21 days [15]. To the best of our knowledge, this is the youngest infant reported to have survived bi-directional Glenn palliation in a standalone case report from India. The clinical value of our report lies in the detailed documentation of primary Glenn palliation in a markedly underweight infant aged 102 days and weighing 2.7 kg, with survival to discharge and documented shunt patency during the available 18-month follow-up. The proposed addition would highlight this contribution.

## Conclusions

This case demonstrates successful technical performance of a bi-directional Glenn shunt in one selected 102-day-old infant weighing 2.7 kg, with discharge on postoperative day 11 and documented anastomotic patency during the available 18-month follow-up. A single favorable outcome cannot establish comparative safety, mortality, a generally lower age or weight threshold, independence from hemodynamic risk, reduced cost, or future Fontan success. Larger prospective or multicenter studies with standardized anthropometry, detailed hemodynamics, nutritional assessment, and longitudinal Fontan outcomes are needed before broader recommendations can be made.
